# 4-Chloro-*N*-(2,6-dichloro­phen­yl)benzamide

**DOI:** 10.1107/S160053680902265X

**Published:** 2009-06-20

**Authors:** Miroslav Tokarčík, B. Thimme Gowda, Jozef Kožíšek, B. P. Sowmya, Hartmut Fuess

**Affiliations:** a Faculty of Chemical and Food Technology, Slovak University of Technology, Radlinského 9, SK-812 37 Bratislava, Slovak Republic; b Department of Chemistry, Mangalore University, Mangalagangotri 574 199, Mangalore, India; c Institute of Materials Science, Darmstadt University of Technology, Petersenstrasse 23, D-64287 Darmstadt, Germany

## Abstract

The title compound, C_13_H_8_Cl_3_NO, crystallizes with four mol­ecules in the asymmetric unit. In the mol­ecular structure, the conformations of the central amide –CONH group show a wide range of dihedral angles with respect to the attached aromatic rings (benzoyl and anilino). The dihedral angles between the amide group and the benzoyl ring are 8.1 (3), 4.3 (3), 27.8 (1) and 32.7 (2)° in the four mol­ecules. The amide group is twisted out of the plane of the anilino ring, as shown by the dihedral angles of 85.4 (1), 74.3 (1), 88.1 (1) and 77.6 (1)° in the four mol­ecules. The aromatic rings are oriented at dihedral angles of 86.6 (1), 78.0 (1), 60.3 (1) and 69.8 (1)° in the four mol­ecules. The crystal structure is stabilized *via* inter­molecular N—H⋯O hydrogen bonds, aromatic aromatic inter­actions, short Cl⋯Cl contacts and C—H⋯Cl hydrogen bonds. Inter­molecular hydrogen bonds connect the mol­ecules into two distinct chains running along the *c* axis of the crystal. One mol­ecule forms an inversion dimer in which the main inter­actions are π–π stacking [centroid–centroid distances = 3.749 (1) and 3.760 (1) Å] and a short Cl⋯Cl contact of 3.408 (1) Å.

## Related literature

For the biological activity of benzamide and benzanilide derivatives, see: Glaser (2007 ▶); Pasha *et al.* (2008 ▶); Brunhofer *et al.* (2008 ▶); Calderone *et al.* (2006 ▶); Stauffer *et al.* (2000 ▶); Lindgren *et al.* (2001 ▶). For anion recognition, see: Kang *et al.* (2006 ▶); Sun *et al.* (2009 ▶). For theoretical study of inter­nal rotations, see: Nishikawa *et al.* (2005 ▶). For related structures, see: Bowes *et al.* (2003 ▶); Gowda *et al.* (2003 ▶); Saeed *et al.* (2008 ▶).
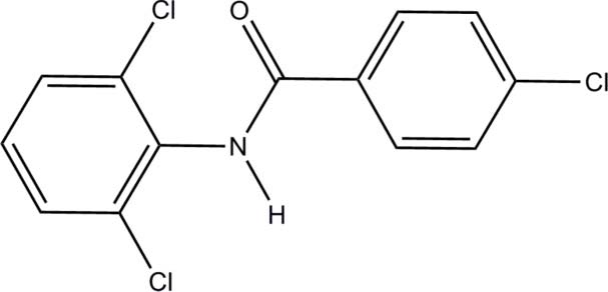


## Experimental

### 

#### Crystal data

C_13_H_8_Cl_3_NO
*M* *_r_* = 300.55
Monoclinic, 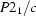

*a* = 16.9411 (3) Å
*b* = 16.3246 (2) Å
*c* = 19.5505 (3) Å
β = 95.3678 (13)°
*V* = 5383.11 (14) Å^3^
*Z* = 16
Mo *K*α radiation
μ = 0.67 mm^−1^
*T* = 295 K
0.51 × 0.11 × 0.09 mm

#### Data collection

Oxford Diffraction Xcalibur diffractometer
Absorption correction: analytical [*CrysAlis RED* (Oxford Diffraction, 2008 ▶), based on Clark & Reid (1995 ▶)] *T*_min_ = 0.754, *T*_max_ = 0.944
180094 measured reflections
10264 independent reflections
5984 reflections with *I* > 2σ(*I*)
*R*_int_ = 0.053

#### Refinement

*R*[*F* ^2^ > 2σ(*F* ^2^)] = 0.041
*wR*(*F* ^2^) = 0.107
*S* = 0.98
10278 reflections
670 parameters
15 restraints
H atoms treated by a mixture of independent and constrained refinement
Δρ_max_ = 0.33 e Å^−3^
Δρ_min_ = −0.23 e Å^−3^

### 

Data collection: *CrysAlis CCD* (Oxford Diffraction, 2008 ▶); cell refinement: *CrysAlis RED* (Oxford Diffraction, 2008 ▶); data reduction: *CrysAlis RED*; program(s) used to solve structure: *SHELXS97* (Sheldrick, 2008 ▶); program(s) used to refine structure: *SHELXL97* (Sheldrick, 2008 ▶); molecular graphics: *ORTEP-3* (Farrugia, 1997 ▶) and *DIAMOND* (Brandenburg, 2001 ▶); software used to prepare material for publication: *SHELXL97*, *PLATON* (Spek, 2009 ▶) and *WinGX* (Farrugia, 1999 ▶).

## Supplementary Material

Crystal structure: contains datablocks global, I. DOI: 10.1107/S160053680902265X/zl2218sup1.cif

Structure factors: contains datablocks I. DOI: 10.1107/S160053680902265X/zl2218Isup2.hkl

Additional supplementary materials:  crystallographic information; 3D view; checkCIF report

## Figures and Tables

**Table 1:** Hydrogen-bond geometry (Å, °)

*D*—H⋯*A*	*D*—H	H⋯*A*	*D*⋯*A*	*D*—H⋯*A*
N1*A*—H1*N*⋯O1*D*^i^	0.855 (16)	2.066 (17)	2.882 (2)	159 (2)
N1*B*—H2*N*⋯O1*C*^ii^	0.816 (15)	2.117 (17)	2.880 (2)	156 (2)
N1*C*—H3*N*⋯O1*B*	0.842 (15)	2.054 (16)	2.875 (2)	165 (2)
N1*D*—H4*N*⋯O1*A*^iii^	0.844 (16)	1.942 (19)	2.728 (2)	154 (2)
C7*A*—H7*A*⋯O1*D*^i^	0.93	2.59	3.489 (3)	164
C10*C*—H10*C*⋯Cl2*B*^iv^	0.93	2.82	3.599 (3)	142

Symmetry codes: (i) 
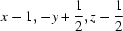
; (ii) 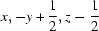; (iii) 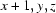; (iv) 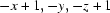.

**Fig. 1.**
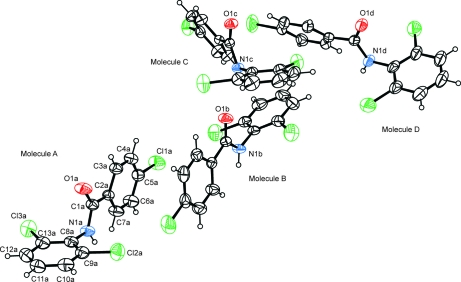
Molecular structure of the title compound showing the atom labelling scheme. Displacement ellipsoids are drawn at the 30% probability level and H atoms are represented as small spheres of arbitrary radii.

**Fig. 2.**
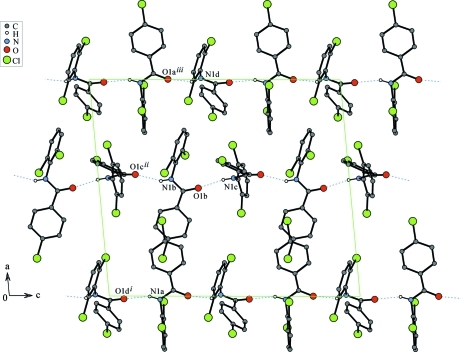
Part of the crystal structure of the title compound, showing the formation of the hydrogen-bonded chains running along the c axis. Symmetry codes: (i) x - 1, -y + 1/2, z - 1/2, (ii) x, -y + 1/2, z - 1/2, (iii) x + 1, y, z. H atoms not involved in hydrogen bonding are omitted.

**Fig. 3.**
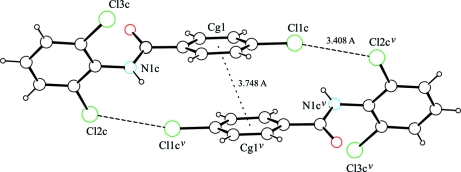
The inversion dimer of the molecule C. The main interaction is π-stacking of benzoyl rings, marked via their centroids Cg1, Cg1(v). Symmetry code: (v) 1 - x, 1 - y, 1 - z. Ring-centroid separation and short Cl···Cl contacts are shown as dashed lines.

## supplementary crystallographic information

### Comment

Various biological activities of benzamide and benzanilide derivatives have been reported in the literature: benzamide based histone deacetylase inhibitors (Glaser 2007), *N*-phenylbenzamides as antimicrobial agents (Pasha *et al.*, 2008), benzanilides with spasmolytic activity (Brunhofer *et al.*, 2008), benzanilide derivatives as effective potassium channel openers (Calderone *et al.*, 2006), *N*-phenyl benzamides as estrogen receptor ligands (Stauffer *et al.*, 2000), *N*-substituted benzamides with immuno-modulatory activity (Lindgren *et al.*, 2001). In supramolecular chemistry, aromatic amides are widely used for anion recognition (Kang *et al.*, 2006). Sun *et al.* (2009) reported nitrophenylbenzamide based chemosensors toward cyanide in aqueous environment. Nishikawa *et al.* (2005) performed DFT-calculations of barriers to internal rotations in aromatic polyamides, including benzanilide.

The title compound, C_13_H_8_Cl_3_NO, (Fig.1), has four unique molecules in the asymmetric unit (further marked as A, B, C and D). In the molecular structure, the conformations of the central amide group CONH with respect to the attached aromatic rings (benzoyl C2/C7, anilino C8/C13) show a wide range of dihedral angle values, which is an indication that the energy of intermolecular interactions is comparable to the barriers of internal rotations. The dihedral angle between the amide group and the benzoyl ring is 8.1 (3), 4.3 (3), 27.8 (1) and 32.7 (2)° in the molecules A, B, C and D, respectively. The amide group is heavily twisted out of the plane of the anilino ring, with dihedral angles of 85.4 (1), 74.3 (1), 88.1 (1), 77.6 (1)° for the molecules A, B, C and D, respectively. This conformation can be attributed to the steric effect of the bulky chloro groups in *ortho* positions. We recall that the corresponding dihedral angle in the parent molecule benzanilide is *ca* 31° (Bowes, *et al.*, 2003). The aromatic rings (benzoyl and anilino) are oriented at dihedral angles of 86.6 (1), 78.0 (1), 60.3 (1) and 69.8 (1)° in the molecules A, B, C and D, respectively. In benzanilide, the aromatic rings make a dihedral angle of *ca* 61°. The bond lengths and bond angles lie within the ranges expected for similar compounds. The endocyclic bond angles in both aromatic rings are sligtly distorted from the ideal value of 120°, reflecting the chloro-substitution effect on the benzene rings. The orientation of an amide group with respect to aromatic rings strongly depends on the local crystal field, which is manifested by significant differences in the torsion angles describing the conformations in the molecules A, B, C and D.

The crystal structure is stabilized by intermolecular N—H···O hydrogen bonds, aromatic aromatic interactions, short Cl···Cl contacts and weak C—H···Cl hydrogen bonds. Intermolecular N—H···O hydrogen bonds connect the molecules into two distinct chains running along the *c* axis of the crystal (Fig. 2). The first chain is linked by hydrogen bonds arising between amidic N, O atoms of the molecules A and D. The second chain is linked by hydrogen bonds arising between amidic N, O atoms of the molecules B and C. The chains are coupled *via* stacking interactions. The two most important π - π stacking formations, which we found using the *PLATON *software (Spek, 2009), are: The stacking between the benzoyl rings of the molecule C at the positions (*x*,*y*,*z*) and (1 - *x*,1 - *y*,1 - *z*). The interplanar distance is 3.538 Å, offset 1.241 Å and ring-centroids separation 3.749 Å. The second stacking is between the anilino rings of the molecule C at the positions (*x*, *y*, *z*) and (1 - *x*, -*y*, 1 - *z*). The interplanar distance is 3.411 Å, offset 1.582 Å and ring-centroids separation 3.760 Å. The molecule C forms an interesting inversion dimer (Fig. 3), with a head-to-tail arrangement. The dimer is stabilized by π - π interaction, a short Cl···Cl contact of 3.408 (1) Å, and possibly dipolar interaction between carbonyl group dipoles. A non-conventional C—H···Cl hydrogen bond (C10*c*—H10*c*···Cl2b) adds to the mosaic of interactions in the crystal structure of the title compound. The H10*c*···Cl2b distance of 2.82 Å is 0.13 Å shorter than the the sum of van der Waals radii for H and Cl.

### Experimental

The title compound was prepared according to the method of Gowda *et al.* (2003). Single crystals used in X-ray diffraction studies were obtained by slow evaporation of its ethanolic solution at room temperature. The purity of the compound was checked by determining its melting point (172–173 °C). The compound was characterized *via* IR and NMR spectroscopy. IR (KBr, cm^-1^): 3271.0 m (N–H stretch), 1654.8 s (C=O stretch), 1299.9 m (C–N stretch). [s = strong band, m = medium band]. ^1^H NMR (CDCl_3_, 300 MHz, p.p.m.): 7.67 (H–N), 7.40 d (H-3,7), 7.23 d (H-11), 7.88 d (H-3,7), 7.46 d (H-4,6). ^13^C NMR (CDCl_3_, 75.5 MHz, p.p.m.): 164.8 (C=O), 132.0 (C-2), 128.9 (C-3,7), 129.1 (C-4,6), 133.7 (C-5), 138.7 (C-8), 131.6 (C-9,13), 128.8 (C-10,12), 128.6 (C-11).

### Refinement

Most of hydrogen atoms were placed in calculated positions with C–H distances in the range 0.93–0.96 Å and constrained to ride on their parent atoms. Amide H atoms were seen in difference maps and were refined with the N–H distances restrained to 0.85 (2) Å. Hydrogen atoms H10, H11 and H12 in the molecule D were refined with C–H distance restrained to 0.94 (3) Å with the aim to remove a Hirschfeld test alert. The *U* values of 8 carbon atoms and 2 chlorine atoms were subject to a rigid bond restraint (DELU command), *i.e.* the components of the displacement parameters in the direction of the bond were restrained to be equal within an effective standard deviation (e.s.d. = 0.007 for atoms C2a, C3a, C8c, C9c, C13*c*, Cl3c, C10d, C11d and e.s.d. = 0.004 for atoms C13d and Cl3d). The *U*_iso_(H) values were set at 1.2*U*_eq_(C-aromatic,*N*).

### Crystal data

**Table:**

C_13_H_8_Cl_3_NO	*F*(000) = 2432
*M**_r_* = 300.55	*D*_x_ = 1.483 Mg m^−^^3^
Monoclinic, *P*2_1_/*c*	Mo *K*α radiation, λ = 0.71073 Å
Hall symbol: -P 2ybc	Cell parameters from 40513 reflections
*a* = 16.9411 (3) Å	θ = 3.0–29.6°
*b* = 16.3246 (2) Å	µ = 0.67 mm^−^^1^
*c* = 19.5505 (3) Å	*T* = 295 K
β = 95.3678 (13)°	Needle, colourless
*V* = 5383.11 (14) Å^3^	0.51 × 0.11 × 0.09 mm
*Z* = 16	

### Data collection

**Table:**

Oxford Diffraction Xcalibur diffractometer	*R*_int_ = 0.053
graphite	θ_max_ = 25.8°, θ_min_ = 2.3°
ω scans with κ offsets	*h* = −20→20
Absorption correction: analytical [*CrysAlis RED* (Oxford Diffraction, 2008), based on Clark & Reid (1995)]	*k* = −19→19
*T*_min_ = 0.754, *T*_max_ = 0.944	*l* = −23→23
180094 measured reflections	3 standard reflections every 120 min
10264 independent reflections	intensity decay: 0.5%
5984 reflections with *I* > 2σ(*I*)	

### Refinement

**Table:**

Refinement on *F*^2^	Primary atom site location: structure-invariant direct methods
Least-squares matrix: full	Secondary atom site location: difference Fourier map
*R*[*F*^2^ > 2σ(*F*^2^)] = 0.041	Hydrogen site location: inferred from neighbouring sites
*wR*(*F*^2^) = 0.107	H atoms treated by a mixture of independent and constrained refinement
*S* = 0.98	*w* = 1/[σ^2^(*F*_o_^2^) + (0.0583*P*)^2^] where *P* = (*F*_o_^2^ + 2*F*_c_^2^)/3
10278 reflections	(Δ/σ)_max_ < 0.001
670 parameters	Δρ_max_ = 0.33 e Å^−^^3^
15 restraints	Δρ_min_ = −0.22 e Å^−^^3^

### Special details

**Table:**

Geometry. All e.s.d.'s (except the e.s.d. in the dihedral angle between two l.s. planes) are estimated using the full covariance matrix. The cell e.s.d.'s are taken into account individually in the estimation of e.s.d.'s in distances, angles and torsion angles; correlations between e.s.d.'s in cell parameters are only used when they are defined by crystal symmetry. An approximate (isotropic) treatment of cell e.s.d.'s is used for estimating e.s.d.'s involving l.s. planes.
Refinement. Refinement of *F*^2^ against ALL reflections. The weighted *R*-factor *wR* and goodness of fit *S* are based on *F*^2^, conventional *R*-factors *R* are based on *F*, with *F* set to zero for negative *F*^2^. The threshold expression of *F*^2^ > σ(*F*^2^) is used only for calculating *R*-factors(gt) *etc*. and is not relevant to the choice of reflections for refinement. *R*-factors based on *F*^2^ are statistically about twice as large as those based on *F*, and *R*- factors based on ALL data will be even larger.

### Fractional atomic coordinates and isotropic or equivalent isotropic displacement parameters (Å^2^)

**Table:**

	*x*	*y*	*z*	*U*_iso_*/*U*_eq_	
N1A	−0.01521 (12)	0.24995 (13)	0.20152 (9)	0.0679 (6)	
H1N	−0.0020 (14)	0.2564 (15)	0.1607 (9)	0.081*	
O1A	0.00098 (11)	0.27519 (12)	0.31328 (7)	0.0872 (6)	
Cl1A	0.34739 (5)	0.42659 (5)	0.23734 (4)	0.1008 (3)	
Cl2A	−0.02618 (5)	0.06753 (5)	0.21458 (4)	0.1059 (3)	
Cl3A	−0.14461 (5)	0.37320 (5)	0.19803 (4)	0.1009 (3)	
C1A	0.02724 (13)	0.27901 (14)	0.25719 (10)	0.0578 (6)	
C2A	0.10565 (13)	0.31648 (13)	0.24908 (9)	0.0517 (5)	
C3A	0.15350 (15)	0.33685 (15)	0.30778 (11)	0.0658 (6)	
H3A	0.1354	0.3275	0.3505	0.079*	
C4A	0.22717 (16)	0.37056 (15)	0.30429 (12)	0.0711 (7)	
H4A	0.2586	0.3838	0.3443	0.085*	
C5A	0.25378 (14)	0.38453 (14)	0.24193 (12)	0.0643 (6)	
C6A	0.20812 (16)	0.36548 (17)	0.18329 (12)	0.0800 (8)	
H6A	0.2264	0.3757	0.1407	0.096*	
C7A	0.13480 (15)	0.33111 (16)	0.18723 (11)	0.0735 (7)	
H7A	0.1041	0.3174	0.1469	0.088*	
C8A	−0.09215 (14)	0.21751 (17)	0.20417 (10)	0.0627 (6)	
C9A	−0.10499 (15)	0.13470 (18)	0.20852 (11)	0.0702 (7)	
C10A	−0.18105 (19)	0.10314 (19)	0.20735 (12)	0.0800 (8)	
H10A	−0.1888	0.047	0.2109	0.096*	
C11A	−0.24487 (17)	0.1553 (2)	0.20087 (12)	0.0820 (8)	
H11A	−0.296	0.1342	0.1987	0.098*	
C12A	−0.23399 (16)	0.2376 (2)	0.19764 (11)	0.0807 (8)	
H12A	−0.2775	0.2726	0.1939	0.097*	
C13A	−0.15816 (16)	0.26914 (17)	0.19990 (10)	0.0712 (7)	
N1B	0.54004 (11)	0.25961 (12)	0.28954 (8)	0.0578 (5)	
H2N	0.5318 (13)	0.2534 (14)	0.2481 (8)	0.069*	
O1B	0.49853 (9)	0.25248 (11)	0.39413 (7)	0.0762 (5)	
Cl1B	0.17328 (5)	0.09853 (6)	0.22580 (6)	0.1275 (3)	
Cl2B	0.65068 (5)	0.13398 (5)	0.35411 (4)	0.1043 (3)	
Cl3B	0.56847 (5)	0.43598 (5)	0.26545 (4)	0.1017 (3)	
C1B	0.48504 (13)	0.24196 (13)	0.33249 (10)	0.0517 (5)	
C2B	0.40793 (12)	0.20796 (12)	0.30239 (10)	0.0484 (5)	
C3B	0.35060 (15)	0.19335 (15)	0.34661 (12)	0.0658 (6)	
H3B	0.3606	0.2064	0.3929	0.079*	
C4B	0.27899 (16)	0.15970 (16)	0.32283 (16)	0.0787 (7)	
H4B	0.2407	0.1497	0.353	0.094*	
C5B	0.26417 (15)	0.14095 (15)	0.25493 (17)	0.0753 (7)	
C6B	0.31894 (17)	0.15589 (18)	0.21067 (14)	0.0841 (8)	
H6B	0.308	0.1438	0.1643	0.101*	
C7B	0.39097 (15)	0.18910 (15)	0.23434 (11)	0.0700 (7)	
H7B	0.4287	0.1989	0.2037	0.084*	
C8B	0.61598 (13)	0.28835 (14)	0.31361 (9)	0.0520 (5)	
C9B	0.67220 (15)	0.23618 (16)	0.34592 (11)	0.0665 (6)	
C10B	0.74665 (17)	0.2637 (2)	0.36897 (13)	0.0840 (8)	
H10B	0.7835	0.2283	0.3912	0.101*	
C11B	0.76574 (18)	0.3435 (2)	0.35883 (14)	0.0883 (9)	
H11B	0.8162	0.3621	0.374	0.106*	
C12B	0.71239 (18)	0.39649 (18)	0.32688 (13)	0.0803 (8)	
H12B	0.7263	0.4507	0.3199	0.096*	
C13B	0.63704 (14)	0.36865 (15)	0.30494 (10)	0.0630 (6)	
N1C	0.54579 (11)	0.23320 (10)	0.53835 (8)	0.0508 (4)	
H3N	0.5371 (12)	0.2468 (13)	0.4968 (8)	0.061*	
O1C	0.56197 (9)	0.27476 (9)	0.64809 (7)	0.0632 (4)	
Cl1C	0.62723 (4)	0.63315 (4)	0.49957 (4)	0.0881 (2)	
Cl2C	0.38314 (4)	0.16952 (5)	0.55737 (4)	0.0940 (2)	
Cl3C	0.69772 (5)	0.14330 (5)	0.54692 (4)	0.1055 (3)	
C1C	0.56049 (11)	0.29103 (13)	0.58689 (10)	0.0459 (5)	
C2C	0.57685 (11)	0.37500 (12)	0.56292 (9)	0.0441 (5)	
C3C	0.56156 (13)	0.44075 (14)	0.60366 (10)	0.0588 (6)	
H3C	0.5406	0.4316	0.6453	0.071*	
C4C	0.57648 (14)	0.51957 (15)	0.58425 (12)	0.0656 (6)	
H4C	0.5645	0.5633	0.6119	0.079*	
C5C	0.60899 (12)	0.53327 (13)	0.52411 (11)	0.0554 (6)	
C6C	0.62633 (14)	0.46970 (14)	0.48319 (11)	0.0638 (6)	
H6C	0.6492	0.4795	0.4426	0.077*	
C7C	0.60982 (13)	0.39061 (13)	0.50214 (10)	0.0566 (6)	
H7C	0.621	0.3473	0.4737	0.068*	
C8C	0.54059 (15)	0.14968 (13)	0.55637 (9)	0.0543 (6)	
C9C	0.46937 (16)	0.11342 (14)	0.56777 (11)	0.0653 (6)	
C10C	0.4646 (2)	0.03189 (16)	0.58582 (12)	0.0832 (8)	
H10C	0.416	0.0085	0.593	0.1*	
C11C	0.5325 (3)	−0.01375 (18)	0.59289 (13)	0.0975 (10)	
H11C	0.5298	−0.0685	0.6057	0.117*	
C12C	0.6042 (2)	0.01942 (19)	0.58145 (13)	0.0915 (9)	
H12C	0.6499	−0.0125	0.5859	0.11*	
C13C	0.60779 (16)	0.10095 (15)	0.56319 (11)	0.0683 (6)	
N1D	1.00625 (13)	0.21879 (12)	0.44495 (8)	0.0716 (6)	
H4N	0.9902 (15)	0.2400 (14)	0.4068 (10)	0.086*	
O1D	0.98706 (12)	0.21685 (11)	0.55695 (7)	0.0870 (6)	
Cl1D	0.82750 (6)	0.58103 (5)	0.47748 (5)	0.1224 (3)	
Cl2D	1.17656 (5)	0.21164 (5)	0.49982 (4)	0.1061 (3)	
Cl3D	0.90147 (6)	0.08528 (6)	0.38692 (4)	0.1133 (3)	
C1D	0.98079 (14)	0.25205 (15)	0.50152 (10)	0.0637 (6)	
C2D	0.94263 (13)	0.33420 (15)	0.49198 (10)	0.0574 (6)	
C3D	0.88233 (16)	0.35364 (17)	0.53201 (11)	0.0741 (7)	
H3D	0.866	0.3153	0.5629	0.089*	
C4D	0.84624 (16)	0.42912 (18)	0.52672 (12)	0.0783 (8)	
H4D	0.8052	0.4417	0.5534	0.094*	
C5D	0.87110 (15)	0.48473 (16)	0.48241 (14)	0.0705 (7)	
C6D	0.93043 (15)	0.46719 (15)	0.44139 (12)	0.0681 (6)	
H6D	0.9464	0.506	0.4107	0.082*	
C7D	0.96600 (13)	0.39125 (15)	0.44644 (11)	0.0589 (6)	
H7D	1.0061	0.3786	0.4188	0.071*	
C8D	1.04163 (19)	0.14073 (17)	0.44522 (11)	0.0743 (8)	
C9D	1.12048 (19)	0.12862 (17)	0.47012 (12)	0.0820 (8)	
C10D	1.1547 (2)	0.0528 (2)	0.47083 (15)	0.0975 (10)	
H10D	1.2093 (14)	0.046 (2)	0.4889 (15)	0.117*	
C11D	1.1102 (3)	−0.0126 (2)	0.44464 (17)	0.1084 (12)	
H11D	1.1308 (19)	−0.0659 (15)	0.4433 (16)	0.13*	
C12D	1.0336 (3)	−0.0034 (2)	0.41819 (16)	0.1030 (11)	
H12D	0.9978 (18)	−0.0453 (18)	0.4045 (16)	0.124*	
C13D	0.99924 (19)	0.07337 (19)	0.41871 (12)	0.0846 (8)	

### Atomic displacement parameters (Å^2^)

**Table:**

	*U*^11^	*U*^22^	*U*^33^	*U*^12^	*U*^13^	*U*^23^
N1A	0.0625 (13)	0.1037 (16)	0.0392 (10)	−0.0228 (11)	0.0139 (9)	−0.0045 (10)
O1A	0.0911 (13)	0.1352 (16)	0.0365 (8)	−0.0286 (11)	0.0115 (8)	0.0063 (9)
Cl1A	0.0782 (5)	0.1158 (6)	0.1075 (5)	−0.0385 (4)	0.0039 (4)	−0.0079 (4)
Cl2A	0.0891 (6)	0.1078 (6)	0.1235 (6)	0.0028 (4)	0.0240 (4)	−0.0029 (5)
Cl3A	0.1086 (6)	0.0957 (6)	0.1020 (5)	−0.0071 (4)	0.0295 (4)	0.0193 (4)
C1A	0.0626 (16)	0.0715 (16)	0.0393 (12)	−0.0033 (12)	0.0053 (11)	0.0076 (10)
C2A	0.0569 (14)	0.0571 (14)	0.0402 (11)	0.0003 (11)	0.0006 (10)	0.0020 (9)
C3A	0.0697 (18)	0.0791 (17)	0.0480 (13)	−0.0042 (14)	0.0024 (11)	0.0017 (11)
C4A	0.0731 (19)	0.0763 (18)	0.0609 (15)	−0.0083 (14)	−0.0096 (13)	−0.0054 (12)
C5A	0.0612 (16)	0.0586 (15)	0.0724 (16)	−0.0085 (12)	0.0024 (13)	−0.0040 (12)
C6A	0.0740 (19)	0.108 (2)	0.0592 (15)	−0.0269 (16)	0.0118 (13)	−0.0024 (13)
C7A	0.0689 (17)	0.104 (2)	0.0466 (13)	−0.0254 (15)	0.0023 (11)	−0.0026 (12)
C8A	0.0577 (17)	0.095 (2)	0.0373 (11)	−0.0174 (15)	0.0132 (10)	0.0008 (11)
C9A	0.0646 (18)	0.092 (2)	0.0552 (14)	−0.0106 (15)	0.0143 (11)	−0.0032 (13)
C10A	0.085 (2)	0.092 (2)	0.0653 (15)	−0.0261 (19)	0.0156 (14)	−0.0031 (13)
C11A	0.0604 (19)	0.122 (3)	0.0647 (16)	−0.0252 (19)	0.0140 (13)	0.0002 (16)
C12A	0.0644 (19)	0.117 (3)	0.0627 (15)	−0.0042 (17)	0.0148 (12)	0.0089 (15)
C13A	0.0693 (19)	0.098 (2)	0.0487 (13)	−0.0138 (16)	0.0171 (11)	0.0058 (12)
N1B	0.0569 (12)	0.0811 (14)	0.0355 (9)	−0.0187 (10)	0.0055 (9)	−0.0068 (9)
O1B	0.0692 (11)	0.1225 (15)	0.0375 (9)	−0.0120 (10)	0.0079 (7)	−0.0091 (8)
Cl1B	0.0605 (5)	0.1105 (7)	0.2077 (10)	−0.0205 (4)	−0.0065 (5)	−0.0284 (6)
Cl2B	0.1170 (7)	0.0748 (5)	0.1224 (6)	0.0019 (4)	0.0172 (5)	0.0197 (4)
Cl3B	0.1091 (6)	0.0838 (5)	0.1127 (5)	0.0075 (4)	0.0135 (5)	0.0211 (4)
C1B	0.0559 (14)	0.0576 (14)	0.0418 (12)	0.0010 (11)	0.0066 (10)	−0.0015 (10)
C2B	0.0500 (13)	0.0466 (12)	0.0490 (12)	0.0026 (10)	0.0069 (10)	0.0033 (9)
C3B	0.0592 (17)	0.0748 (17)	0.0650 (14)	0.0039 (13)	0.0139 (12)	0.0040 (12)
C4B	0.0545 (18)	0.0732 (18)	0.112 (2)	0.0007 (14)	0.0272 (15)	0.0118 (16)
C5B	0.0510 (16)	0.0578 (16)	0.116 (2)	−0.0005 (12)	0.0007 (16)	−0.0101 (15)
C6B	0.0687 (19)	0.101 (2)	0.0806 (17)	−0.0128 (16)	−0.0032 (15)	−0.0196 (15)
C7B	0.0613 (17)	0.0889 (19)	0.0600 (14)	−0.0165 (14)	0.0065 (12)	−0.0091 (12)
C8B	0.0546 (15)	0.0651 (16)	0.0367 (10)	−0.0110 (12)	0.0069 (10)	−0.0084 (10)
C9B	0.0646 (17)	0.0793 (17)	0.0552 (13)	−0.0067 (14)	0.0043 (12)	−0.0062 (12)
C10B	0.067 (2)	0.107 (2)	0.0757 (17)	0.0041 (17)	−0.0062 (14)	−0.0021 (15)
C11B	0.0616 (19)	0.123 (3)	0.0793 (18)	−0.0240 (19)	−0.0015 (14)	−0.0253 (18)
C12B	0.085 (2)	0.0799 (19)	0.0788 (17)	−0.0300 (17)	0.0223 (16)	−0.0221 (15)
C13B	0.0664 (17)	0.0700 (17)	0.0537 (13)	−0.0098 (13)	0.0116 (11)	−0.0087 (11)
N1C	0.0691 (12)	0.0469 (11)	0.0359 (9)	0.0005 (9)	0.0015 (8)	0.0018 (8)
O1C	0.0834 (11)	0.0681 (10)	0.0381 (8)	−0.0007 (8)	0.0063 (7)	0.0022 (7)
Cl1C	0.0757 (5)	0.0547 (4)	0.1344 (6)	−0.0027 (3)	0.0129 (4)	0.0078 (4)
Cl2C	0.0776 (5)	0.0817 (5)	0.1262 (6)	0.0045 (4)	0.0272 (4)	0.0300 (4)
Cl3C	0.0733 (5)	0.1196 (7)	0.1222 (6)	0.0188 (5)	0.0019 (4)	−0.0090 (5)
C1C	0.0435 (13)	0.0566 (14)	0.0376 (12)	0.0071 (10)	0.0036 (9)	−0.0021 (10)
C2C	0.0412 (12)	0.0520 (13)	0.0379 (10)	0.0028 (9)	−0.0020 (9)	−0.0048 (9)
C3C	0.0686 (16)	0.0580 (16)	0.0507 (12)	−0.0006 (12)	0.0103 (11)	−0.0080 (11)
C4C	0.0674 (16)	0.0563 (16)	0.0736 (16)	0.0035 (12)	0.0087 (12)	−0.0192 (12)
C5C	0.0429 (13)	0.0490 (14)	0.0728 (15)	−0.0009 (10)	−0.0029 (11)	0.0017 (11)
C6C	0.0750 (17)	0.0552 (16)	0.0639 (14)	−0.0062 (13)	0.0208 (12)	0.0013 (12)
C7C	0.0664 (16)	0.0504 (14)	0.0546 (13)	0.0009 (11)	0.0143 (11)	−0.0089 (10)
C8C	0.0742 (16)	0.0479 (14)	0.0405 (11)	0.0069 (12)	0.0031 (10)	−0.0007 (9)
C9C	0.0870 (18)	0.0528 (15)	0.0570 (13)	0.0039 (13)	0.0107 (12)	0.0090 (11)
C10C	0.116 (2)	0.0564 (18)	0.0794 (17)	−0.0065 (17)	0.0226 (16)	0.0112 (13)
C11C	0.166 (4)	0.0521 (18)	0.0757 (18)	0.017 (2)	0.018 (2)	0.0114 (13)
C12C	0.129 (3)	0.068 (2)	0.0773 (18)	0.040 (2)	0.0069 (18)	0.0011 (15)
C13C	0.0857 (18)	0.0654 (17)	0.0529 (13)	0.0140 (14)	0.0008 (12)	−0.0059 (11)
N1D	0.1072 (17)	0.0729 (14)	0.0357 (10)	0.0363 (12)	0.0129 (10)	0.0074 (9)
O1D	0.1234 (16)	0.1011 (13)	0.0381 (8)	0.0518 (12)	0.0168 (8)	0.0132 (8)
Cl1D	0.1094 (7)	0.0829 (6)	0.1709 (8)	0.0390 (5)	−0.0071 (6)	−0.0135 (5)
Cl2D	0.1100 (6)	0.1067 (6)	0.1018 (5)	0.0202 (5)	0.0107 (4)	0.0049 (4)
Cl3D	0.1341 (8)	0.1160 (7)	0.0871 (5)	0.0134 (5)	−0.0045 (5)	−0.0027 (4)
C1D	0.0775 (17)	0.0775 (17)	0.0357 (12)	0.0244 (13)	0.0039 (11)	−0.0003 (11)
C2D	0.0624 (15)	0.0749 (16)	0.0336 (10)	0.0183 (12)	−0.0028 (10)	−0.0047 (11)
C3D	0.0843 (19)	0.093 (2)	0.0461 (12)	0.0298 (16)	0.0099 (12)	0.0066 (12)
C4D	0.0760 (19)	0.102 (2)	0.0575 (14)	0.0329 (17)	0.0070 (13)	−0.0023 (15)
C5D	0.0596 (17)	0.0672 (17)	0.0802 (17)	0.0184 (13)	−0.0173 (14)	−0.0169 (14)
C6D	0.0598 (16)	0.0634 (17)	0.0788 (16)	−0.0047 (13)	−0.0056 (13)	−0.0020 (12)
C7D	0.0469 (14)	0.0691 (16)	0.0599 (13)	0.0048 (12)	0.0010 (10)	−0.0093 (12)
C8D	0.116 (2)	0.0710 (19)	0.0386 (12)	0.0345 (18)	0.0239 (13)	0.0051 (12)
C9D	0.109 (2)	0.080 (2)	0.0606 (15)	0.0398 (18)	0.0284 (15)	0.0092 (13)
C10D	0.118 (3)	0.100 (3)	0.0791 (19)	0.048 (2)	0.0332 (18)	0.0100 (17)
C11D	0.160 (4)	0.083 (2)	0.088 (2)	0.058 (2)	0.042 (2)	0.0066 (18)
C12D	0.151 (4)	0.082 (3)	0.080 (2)	0.024 (2)	0.028 (2)	−0.0053 (16)
C13D	0.120 (2)	0.086 (2)	0.0501 (14)	0.0349 (19)	0.0186 (14)	0.0024 (14)

### Geometric parameters (Å, °)

**Table:**

N1A—C1A	1.334 (3)	N1C—C1C	1.345 (2)
N1A—C8A	1.412 (3)	N1C—C8C	1.413 (3)
N1A—H1N	0.855 (16)	N1C—H3N	0.842 (15)
O1A—C1A	1.223 (2)	O1C—C1C	1.224 (2)
Cl1A—C5A	1.738 (2)	Cl1C—C5C	1.736 (2)
Cl2A—C9A	1.723 (3)	Cl2C—C9C	1.720 (3)
Cl3A—C13A	1.715 (3)	Cl3C—C13C	1.729 (3)
C1A—C2A	1.484 (3)	C1C—C2C	1.483 (3)
C2A—C7A	1.369 (3)	C2C—C3C	1.376 (3)
C2A—C3A	1.382 (3)	C2C—C7C	1.383 (3)
C3A—C4A	1.371 (3)	C3C—C4C	1.372 (3)
C3A—H3A	0.93	C3C—H3C	0.93
C4A—C5A	1.358 (3)	C4C—C5C	1.363 (3)
C4A—H4A	0.93	C4C—H4C	0.93
C5A—C6A	1.358 (3)	C5C—C6C	1.359 (3)
C6A—C7A	1.372 (3)	C6C—C7C	1.379 (3)
C6A—H6A	0.93	C6C—H6C	0.93
C7A—H7A	0.93	C7C—H7C	0.93
C8A—C9A	1.373 (3)	C8C—C9C	1.381 (3)
C8A—C13A	1.397 (4)	C8C—C13C	1.385 (3)
C9A—C10A	1.386 (4)	C9C—C10C	1.381 (3)
C10A—C11A	1.373 (4)	C10C—C11C	1.366 (4)
C10A—H10A	0.93	C10C—H10C	0.93
C11A—C12A	1.358 (4)	C11C—C12C	1.368 (4)
C11A—H11A	0.93	C11C—H11C	0.93
C12A—C13A	1.381 (4)	C12C—C13C	1.381 (4)
C12A—H12A	0.93	C12C—H12C	0.93
N1B—C1B	1.343 (3)	N1D—C1D	1.339 (3)
N1B—C8B	1.408 (3)	N1D—C8D	1.408 (3)
N1B—H2N	0.816 (15)	N1D—H4N	0.844 (16)
O1B—C1B	1.218 (2)	O1D—C1D	1.222 (2)
Cl1B—C5B	1.735 (3)	Cl1D—C5D	1.736 (2)
Cl2B—C9B	1.718 (3)	Cl2D—C9D	1.724 (3)
Cl3B—C13B	1.728 (3)	Cl3D—C13D	1.725 (3)
C1B—C2B	1.489 (3)	C1D—C2D	1.493 (3)
C2B—C7B	1.370 (3)	C2D—C7D	1.372 (3)
C2B—C3B	1.380 (3)	C2D—C3D	1.381 (3)
C3B—C4B	1.373 (4)	C3D—C4D	1.375 (3)
C3B—H3B	0.93	C3D—H3D	0.93
C4B—C5B	1.363 (4)	C4D—C5D	1.349 (4)
C4B—H4B	0.93	C4D—H4D	0.93
C5B—C6B	1.349 (4)	C5D—C6D	1.373 (3)
C6B—C7B	1.375 (3)	C6D—C7D	1.378 (3)
C6B—H6B	0.93	C6D—H6D	0.93
C7B—H7B	0.93	C7D—H7D	0.93
C8B—C13B	1.373 (3)	C8D—C13D	1.387 (4)
C8B—C9B	1.385 (3)	C8D—C9D	1.392 (4)
C9B—C10B	1.375 (3)	C9D—C10D	1.366 (4)
C10B—C11B	1.360 (4)	C10D—C11D	1.378 (5)
C10B—H10B	0.93	C10D—H10D	0.96 (2)
C11B—C12B	1.361 (4)	C11D—C12D	1.360 (5)
C11B—H11B	0.93	C11D—H11D	0.94 (2)
C12B—C13B	1.385 (3)	C12D—C13D	1.382 (4)
C12B—H12B	0.93	C12D—H12D	0.94 (2)
			
C1A—N1A—C8A	122.16 (17)	C1C—N1C—C8C	120.87 (16)
C1A—N1A—H1N	123.3 (17)	C1C—N1C—H3N	119.9 (15)
C8A—N1A—H1N	113.8 (17)	C8C—N1C—H3N	119.1 (15)
O1A—C1A—N1A	120.0 (2)	O1C—C1C—N1C	121.53 (19)
O1A—C1A—C2A	121.40 (19)	O1C—C1C—C2C	121.48 (17)
N1A—C1A—C2A	118.61 (18)	N1C—C1C—C2C	116.97 (16)
C7A—C2A—C3A	117.3 (2)	C3C—C2C—C7C	117.8 (2)
C7A—C2A—C1A	124.51 (19)	C3C—C2C—C1C	119.17 (18)
C3A—C2A—C1A	118.14 (19)	C7C—C2C—C1C	122.96 (18)
C4A—C3A—C2A	121.4 (2)	C4C—C3C—C2C	121.5 (2)
C4A—C3A—H3A	119.3	C4C—C3C—H3C	119.3
C2A—C3A—H3A	119.3	C2C—C3C—H3C	119.3
C5A—C4A—C3A	119.5 (2)	C5C—C4C—C3C	119.5 (2)
C5A—C4A—H4A	120.3	C5C—C4C—H4C	120.3
C3A—C4A—H4A	120.3	C3C—C4C—H4C	120.3
C6A—C5A—C4A	120.6 (2)	C6C—C5C—C4C	120.6 (2)
C6A—C5A—Cl1A	119.8 (2)	C6C—C5C—Cl1C	120.05 (18)
C4A—C5A—Cl1A	119.59 (19)	C4C—C5C—Cl1C	119.30 (18)
C5A—C6A—C7A	119.6 (2)	C5C—C6C—C7C	119.7 (2)
C5A—C6A—H6A	120.2	C5C—C6C—H6C	120.1
C7A—C6A—H6A	120.2	C7C—C6C—H6C	120.1
C2A—C7A—C6A	121.6 (2)	C6C—C7C—C2C	120.78 (19)
C2A—C7A—H7A	119.2	C6C—C7C—H7C	119.6
C6A—C7A—H7A	119.2	C2C—C7C—H7C	119.6
C9A—C8A—C13A	117.9 (2)	C9C—C8C—C13C	117.5 (2)
C9A—C8A—N1A	121.6 (2)	C9C—C8C—N1C	122.0 (2)
C13A—C8A—N1A	120.5 (2)	C13C—C8C—N1C	120.6 (2)
C8A—C9A—C10A	121.1 (3)	C8C—C9C—C10C	121.8 (2)
C8A—C9A—Cl2A	120.3 (2)	C8C—C9C—Cl2C	119.87 (17)
C10A—C9A—Cl2A	118.5 (2)	C10C—C9C—Cl2C	118.3 (2)
C11A—C10A—C9A	119.6 (3)	C11C—C10C—C9C	118.8 (3)
C11A—C10A—H10A	120.2	C11C—C10C—H10C	120.6
C9A—C10A—H10A	120.2	C9C—C10C—H10C	120.6
C12A—C11A—C10A	120.6 (3)	C10C—C11C—C12C	121.3 (3)
C12A—C11A—H11A	119.7	C10C—C11C—H11C	119.3
C10A—C11A—H11A	119.7	C12C—C11C—H11C	119.3
C11A—C12A—C13A	119.8 (3)	C11C—C12C—C13C	119.0 (3)
C11A—C12A—H12A	120.1	C11C—C12C—H12C	120.5
C13A—C12A—H12A	120.1	C13C—C12C—H12C	120.5
C12A—C13A—C8A	121.0 (3)	C12C—C13C—C8C	121.5 (3)
C12A—C13A—Cl3A	119.6 (2)	C12C—C13C—Cl3C	119.6 (2)
C8A—C13A—Cl3A	119.4 (2)	C8C—C13C—Cl3C	118.9 (2)
C1B—N1B—C8B	121.88 (16)	C1D—N1D—C8D	122.27 (18)
C1B—N1B—H2N	122.1 (16)	C1D—N1D—H4N	117.4 (17)
C8B—N1B—H2N	116.1 (16)	C8D—N1D—H4N	118.5 (17)
O1B—C1B—N1B	120.78 (19)	O1D—C1D—N1D	122.2 (2)
O1B—C1B—C2B	121.35 (19)	O1D—C1D—C2D	122.27 (19)
N1B—C1B—C2B	117.86 (17)	N1D—C1D—C2D	115.48 (18)
C7B—C2B—C3B	118.3 (2)	C7D—C2D—C3D	119.0 (2)
C7B—C2B—C1B	124.35 (19)	C7D—C2D—C1D	122.9 (2)
C3B—C2B—C1B	117.37 (18)	C3D—C2D—C1D	118.1 (2)
C4B—C3B—C2B	120.6 (2)	C4D—C3D—C2D	120.7 (2)
C4B—C3B—H3B	119.7	C4D—C3D—H3D	119.6
C2B—C3B—H3B	119.7	C2D—C3D—H3D	119.6
C5B—C4B—C3B	119.8 (2)	C5D—C4D—C3D	119.2 (2)
C5B—C4B—H4B	120.1	C5D—C4D—H4D	120.4
C3B—C4B—H4B	120.1	C3D—C4D—H4D	120.4
C6B—C5B—C4B	120.6 (2)	C4D—C5D—C6D	121.6 (2)
C6B—C5B—Cl1B	120.3 (2)	C4D—C5D—Cl1D	119.2 (2)
C4B—C5B—Cl1B	119.1 (2)	C6D—C5D—Cl1D	119.2 (2)
C5B—C6B—C7B	119.8 (2)	C5D—C6D—C7D	119.0 (2)
C5B—C6B—H6B	120.1	C5D—C6D—H6D	120.5
C7B—C6B—H6B	120.1	C7D—C6D—H6D	120.5
C2B—C7B—C6B	121.0 (2)	C2D—C7D—C6D	120.4 (2)
C2B—C7B—H7B	119.5	C2D—C7D—H7D	119.8
C6B—C7B—H7B	119.5	C6D—C7D—H7D	119.8
C13B—C8B—C9B	117.9 (2)	C13D—C8D—C9D	117.7 (2)
C13B—C8B—N1B	121.1 (2)	C13D—C8D—N1D	120.7 (3)
C9B—C8B—N1B	121.0 (2)	C9D—C8D—N1D	121.6 (3)
C10B—C9B—C8B	121.3 (2)	C10D—C9D—C8D	121.6 (3)
C10B—C9B—Cl2B	118.9 (2)	C10D—C9D—Cl2D	119.2 (3)
C8B—C9B—Cl2B	119.77 (19)	C8D—C9D—Cl2D	119.1 (2)
C11B—C10B—C9B	119.3 (3)	C9D—C10D—C11D	118.8 (3)
C11B—C10B—H10B	120.4	C9D—C10D—H10D	120 (2)
C9B—C10B—H10B	120.4	C11D—C10D—H10D	121 (2)
C10B—C11B—C12B	121.3 (3)	C12D—C11D—C10D	121.6 (3)
C10B—C11B—H11B	119.4	C12D—C11D—H11D	116 (2)
C12B—C11B—H11B	119.4	C10D—C11D—H11D	123 (2)
C11B—C12B—C13B	119.1 (3)	C11D—C12D—C13D	119.1 (3)
C11B—C12B—H12B	120.4	C11D—C12D—H12D	127 (2)
C13B—C12B—H12B	120.4	C13D—C12D—H12D	114 (2)
C8B—C13B—C12B	121.2 (2)	C12D—C13D—C8D	121.2 (3)
C8B—C13B—Cl3B	119.39 (19)	C12D—C13D—Cl3D	119.4 (3)
C12B—C13B—Cl3B	119.4 (2)	C8D—C13D—Cl3D	119.4 (2)
			
C8A—N1A—C1A—O1A	2.8 (4)	C8C—N1C—C1C—O1C	−7.4 (3)
C8A—N1A—C1A—C2A	−176.4 (2)	C8C—N1C—C1C—C2C	171.07 (18)
O1A—C1A—C2A—C7A	−172.4 (2)	O1C—C1C—C2C—C3C	−27.0 (3)
N1A—C1A—C2A—C7A	6.9 (4)	N1C—C1C—C2C—C3C	154.53 (19)
O1A—C1A—C2A—C3A	8.9 (3)	O1C—C1C—C2C—C7C	150.5 (2)
N1A—C1A—C2A—C3A	−171.9 (2)	N1C—C1C—C2C—C7C	−28.0 (3)
C7A—C2A—C3A—C4A	0.2 (4)	C7C—C2C—C3C—C4C	1.6 (3)
C1A—C2A—C3A—C4A	179.0 (2)	C1C—C2C—C3C—C4C	179.2 (2)
C2A—C3A—C4A—C5A	0.2 (4)	C2C—C3C—C4C—C5C	−1.6 (3)
C3A—C4A—C5A—C6A	0.0 (4)	C3C—C4C—C5C—C6C	0.3 (3)
C3A—C4A—C5A—Cl1A	−179.6 (2)	C3C—C4C—C5C—Cl1C	179.65 (17)
C4A—C5A—C6A—C7A	−0.6 (4)	C4C—C5C—C6C—C7C	1.0 (3)
Cl1A—C5A—C6A—C7A	179.0 (2)	Cl1C—C5C—C6C—C7C	−178.36 (17)
C3A—C2A—C7A—C6A	−0.8 (4)	C5C—C6C—C7C—C2C	−1.0 (3)
C1A—C2A—C7A—C6A	−179.5 (2)	C3C—C2C—C7C—C6C	−0.3 (3)
C5A—C6A—C7A—C2A	1.0 (4)	C1C—C2C—C7C—C6C	−177.8 (2)
C1A—N1A—C8A—C9A	−97.7 (3)	C1C—N1C—C8C—C9C	91.7 (2)
C1A—N1A—C8A—C13A	84.6 (3)	C1C—N1C—C8C—C13C	−88.3 (2)
C13A—C8A—C9A—C10A	1.3 (3)	C13C—C8C—C9C—C10C	0.5 (3)
N1A—C8A—C9A—C10A	−176.47 (19)	N1C—C8C—C9C—C10C	−179.56 (19)
C13A—C8A—C9A—Cl2A	−179.50 (15)	C13C—C8C—C9C—Cl2C	−177.83 (15)
N1A—C8A—C9A—Cl2A	2.8 (3)	N1C—C8C—C9C—Cl2C	2.1 (3)
C8A—C9A—C10A—C11A	0.8 (3)	C8C—C9C—C10C—C11C	0.4 (4)
Cl2A—C9A—C10A—C11A	−178.44 (18)	Cl2C—C9C—C10C—C11C	178.7 (2)
C9A—C10A—C11A—C12A	−1.9 (4)	C9C—C10C—C11C—C12C	−1.0 (4)
C10A—C11A—C12A—C13A	0.9 (4)	C10C—C11C—C12C—C13C	0.8 (4)
C11A—C12A—C13A—C8A	1.2 (3)	C11C—C12C—C13C—C8C	0.1 (4)
C11A—C12A—C13A—Cl3A	−178.34 (18)	C11C—C12C—C13C—Cl3C	−178.7 (2)
C9A—C8A—C13A—C12A	−2.3 (3)	C9C—C8C—C13C—C12C	−0.7 (3)
N1A—C8A—C13A—C12A	175.46 (19)	N1C—C8C—C13C—C12C	179.31 (19)
C9A—C8A—C13A—Cl3A	177.28 (16)	C9C—C8C—C13C—Cl3C	178.16 (16)
N1A—C8A—C13A—Cl3A	−5.0 (3)	N1C—C8C—C13C—Cl3C	−1.8 (3)
C8B—N1B—C1B—O1B	2.0 (3)	C8D—N1D—C1D—O1D	0.4 (4)
C8B—N1B—C1B—C2B	−176.97 (19)	C8D—N1D—C1D—C2D	−178.3 (3)
O1B—C1B—C2B—C7B	−175.1 (2)	O1D—C1D—C2D—C7D	147.4 (2)
N1B—C1B—C2B—C7B	3.9 (3)	N1D—C1D—C2D—C7D	−33.9 (3)
O1B—C1B—C2B—C3B	3.7 (3)	O1D—C1D—C2D—C3D	−31.2 (3)
N1B—C1B—C2B—C3B	−177.3 (2)	N1D—C1D—C2D—C3D	147.5 (2)
C7B—C2B—C3B—C4B	0.9 (3)	C7D—C2D—C3D—C4D	−0.3 (3)
C1B—C2B—C3B—C4B	−178.0 (2)	C1D—C2D—C3D—C4D	178.4 (2)
C2B—C3B—C4B—C5B	−0.3 (4)	C2D—C3D—C4D—C5D	−0.8 (4)
C3B—C4B—C5B—C6B	−0.7 (4)	C3D—C4D—C5D—C6D	1.4 (4)
C3B—C4B—C5B—Cl1B	−180.0 (2)	C3D—C4D—C5D—Cl1D	−178.18 (19)
C4B—C5B—C6B—C7B	1.1 (4)	C4D—C5D—C6D—C7D	−0.9 (4)
Cl1B—C5B—C6B—C7B	−179.6 (2)	Cl1D—C5D—C6D—C7D	178.70 (16)
C3B—C2B—C7B—C6B	−0.5 (4)	C3D—C2D—C7D—C6D	0.8 (3)
C1B—C2B—C7B—C6B	178.3 (2)	C1D—C2D—C7D—C6D	−177.8 (2)
C5B—C6B—C7B—C2B	−0.5 (4)	C5D—C6D—C7D—C2D	−0.3 (3)
C1B—N1B—C8B—C13B	−107.0 (2)	C1D—N1D—C8D—C13D	102.9 (3)
C1B—N1B—C8B—C9B	73.9 (3)	C1D—N1D—C8D—C9D	−78.9 (3)
C13B—C8B—C9B—C10B	0.3 (3)	C13D—C8D—C9D—C10D	−2.2 (4)
N1B—C8B—C9B—C10B	179.4 (2)	N1D—C8D—C9D—C10D	179.6 (2)
C13B—C8B—C9B—Cl2B	−176.64 (16)	C13D—C8D—C9D—Cl2D	177.01 (17)
N1B—C8B—C9B—Cl2B	2.4 (3)	N1D—C8D—C9D—Cl2D	−1.2 (3)
C8B—C9B—C10B—C11B	−1.0 (4)	C8D—C9D—C10D—C11D	1.6 (4)
Cl2B—C9B—C10B—C11B	175.9 (2)	Cl2D—C9D—C10D—C11D	−177.6 (2)
C9B—C10B—C11B—C12B	0.6 (4)	C9D—C10D—C11D—C12D	0.1 (5)
C10B—C11B—C12B—C13B	0.6 (4)	C10D—C11D—C12D—C13D	−1.1 (5)
C9B—C8B—C13B—C12B	0.9 (3)	C11D—C12D—C13D—C8D	0.4 (4)
N1B—C8B—C13B—C12B	−178.19 (19)	C11D—C12D—C13D—Cl3D	−178.7 (2)
C9B—C8B—C13B—Cl3B	−179.54 (15)	C9D—C8D—C13D—C12D	1.2 (4)
N1B—C8B—C13B—Cl3B	1.4 (3)	N1D—C8D—C13D—C12D	179.4 (2)
C11B—C12B—C13B—C8B	−1.4 (3)	C9D—C8D—C13D—Cl3D	−179.74 (17)
C11B—C12B—C13B—Cl3B	179.1 (2)	N1D—C8D—C13D—Cl3D	−1.5 (3)

### Hydrogen-bond geometry (Å, °)

**Table:**

*D*—H···*A*	*D*—H	H···*A*	*D*···*A*	*D*—H···*A*
N1A—H1N···O1D^i^	0.86 (2)	2.07 (2)	2.882 (2)	159 (2)
N1B—H2N···O1C^ii^	0.82 (2)	2.12 (2)	2.880 (2)	156 (2)
N1C—H3N···O1B	0.84 (2)	2.05 (2)	2.875 (2)	165 (2)
N1D—H4N···O1A^iii^	0.84 (2)	1.94 (2)	2.728 (2)	154 (2)
C7A—H7A···O1D^i^	0.93	2.59	3.489 (3)	164
C10C—H10C···Cl2B^iv^	0.93	2.82	3.599 (3)	142

Symmetry codes: (i) *x*−1, −*y*+1/2, *z*−1/2; (ii) *x*, −*y*+1/2, *z*−1/2; (iii) *x*+1, *y*, *z*; (iv) −*x*+1, −*y*, −*z*+1.
